# Research progress on the proteins involved in African swine fever virus infection and replication

**DOI:** 10.3389/fimmu.2022.947180

**Published:** 2022-07-22

**Authors:** Xianghan Duan, Yi Ru, Wenping Yang, Jingjing Ren, Rongzeng Hao, Xiaodong Qin, Dan Li, Haixue Zheng

**Affiliations:** ^1^ State Key Laboratory of Veterinary Etiological Biology and OIE/National Foot and Mouth Disease Reference Laboratory, Lanzhou Veterinary Research Institute, Chinese Academy of Agricultural Sciences, Lanzhou, China; ^2^ State Key Laboratory of Veterinary Etiological Biology, College of Veterinary Medicine, Lanzhou University, Lanzhou Veterinary Research Institute, Chinese Academy of Agricultural Sciences, Lanzhou, China

**Keywords:** African swine fever virus, infection, replication, virus factory, transcription

## Abstract

African swine fever (ASF) is an acute, hemorrhagic and highly contagious infectious disease caused by African swine fever virus (ASFV), which infects domestic pigs or wild boars. It is characterized by short course of disease, high fever and hemorrhagic lesions, with mortality of up to 100% from acute infection. Up to now, the lack of commercial vaccines and effective drugs has seriously threatened the healthy economic development of the global pig industry. ASFV is a double-stranded DNA virus and genome varies between about 170-194 kb, which encodes 150-200 viral proteins, including 68 structural proteins and more than 100 non-structural proteins. In recent years, although the research on structure and function of ASFV-encoded proteins has been deepened, the structure and infection process of ASFV are still not clear. This review summarizes the main process of ASFV infection, replication and functions of related viral proteins to provide scientific basis and theoretical basis for ASFV research and vaccine development.

## Introduction

African swine fever (ASF) is a highly infectious viral disease of pigs caused by African swine fever virus (ASFV). It has a short course of disease, high morbidity and mortality (1). The World Organization for Animal Health [Office International des épizooties (OIE)] lists ASF as one of the notifiable animal diseases that must be reported. ASF was first diagnosed in Kenya in 1909, then confirmed in 1921, and then broken out in other parts of the African continent (2). The disease was also found in Portugal in 1957, which was the first time that ASF was found outside the African continent. In 2018, the first case of ASF was found in Shenyang, Liaoning Province, China, and then quickly spreader to 23 provinces across the country, caused serious economic losses to the pig industry (3). Today, ASF is rapidly spreading among animals in the world, with a devastating impact on the global pork and feed industries (4).

ASFV is the only member of the family *Asfarviridae*. In addition, it is a large double-stranded DNA arbovirus. Domestic pigs, wild boars, warthogs, bush pigs and soft ticks are hosts of ASFV, and porcine monocytes-macrophages are the main target cells of ASFV (5). The ASFV is an enveloped virus with icosahedral morphology and an average diameter of 200 nm, which is composed of five parts: outer capsule membrane, capsid, inner capsule membrane, core shell and nucleoid. The capsid is composed of one major (p72) and four minor (pM1249L, p17, p49 and pH240R) proteins (1). According to the sequence variation in the C-terminal region encoding the main capsid protein p72, ASFV can be divided into 24 genotypes. The ASFV genome is 170-194 kb in length, has 150-167 open reading frames (ORFs). The genome can encode 150-200 proteins, including 68 structural proteins and more than 100 non-structural proteins (6). Collectively, these studies demonstrate the complex structure and proliferation of ASFV (7).

**Figure 1:**
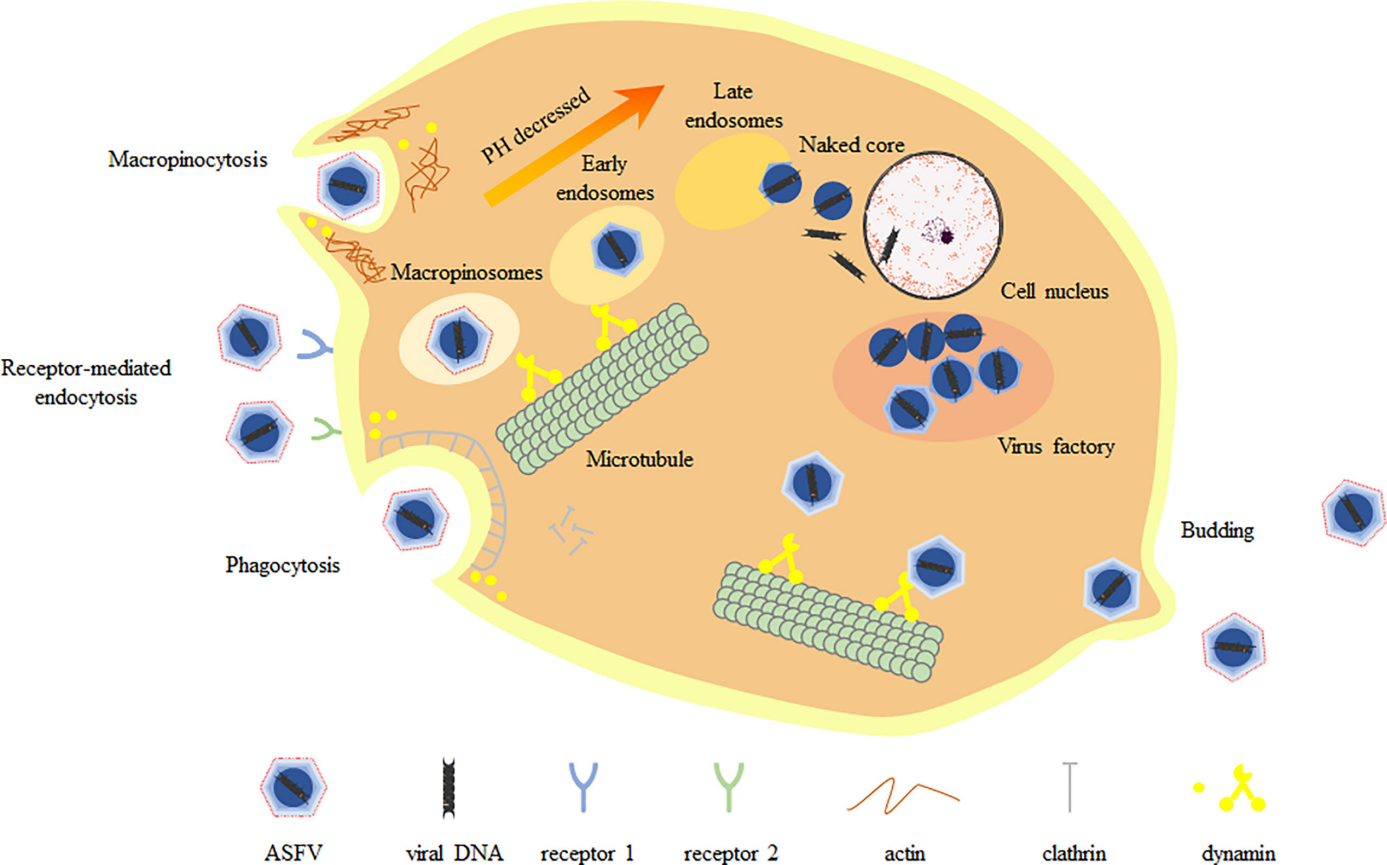
Replication cycle of ASFV. ASFV enters host cells mainly through macropinocytosis and endocytosis, during which the capsid is removed. Endosomes migrate, mature and acidify in the cell, gradually forming multiple vesicles (MVB), late endosomes (LE) and endosomal lysosomes. The fusion of the viral inner membrane with the late endosomal membrane releases the viral core into the cytoplasm. The virus core is routed to the virus factory through microtubule system. Mature virions arrive at the cell surface by microtubule-mediated transporting and finally release from the cell by budding on the plasma membrane.

The virus lacks complete enzyme system and ribosome and does not have the raw materials and energy to synthesize its own components. Therefore, it must invade the susceptible host cells. The process of viral proliferation in host cells is divided into adsorption, penetration, uncoating, biosynthesis and assembly releasing, which is also called the replication cycle (**Figure 1**). Monocytes and porcine alveolar macrophages are the main target cells of ASFV infection (8). ASFV enters the cell mainly through phagocytosis and non-receptor-mediated macropinocytosis, after which the virions are transported by early endosomal, and uncoated by endosomal acidification at late endosomal stage, finally releasing the core containing the viral genome into the cytoplasm. The study found that ASFV genome was detected in the nucleus using BrdU pulse labeling assay at the early stage of infection of porcine mononuclear macrophages (MDMs) and Vero cells (9). As with other viral infections, nuclear recombination can occur during infection to provide an environment for intranuclear replication. In addition, ASFV interacts with subnuclear domains and chromatin structures. ASFV undergoes nuclear recombination, alters subnuclear domains, relocates ataxia telangiectasia mutated rad-3 related factors and promotes heterochromatin (10). These may control transcription, thereby inhibiting host cell gene expression in favor of the virus’s own replication. Viral gene expression is divided into four stages: immediate-early, early, middle and late (11, 12). Only transient replication occurs in the nucleus, and early gene expression products form a virus factory (VF) in the cytoplasm, and it is close to the microtubule center near the nucleus. In VF, the aggregated particles use the membrane of the broken endoplasmic reticulum as a viral precursor (13, 14). After the mature virions in the cytoplasm are gradually assembled and formed, they move to the cell surface through microtubule mediated transport, and then release from the cell by budding on the plasma membrane (15, 16).

## Adsorption and invasion

ASFV, as a large envelope DNA virus, has a complex invasion process, involving a variety of viral proteins (**Table 1**). The ASFV p12, P54, p72 and CD2v (infected red blood cells) structural proteins involved in the process of virus adsorption. The p12 protein allows membrane proteins on the cell surface to act as ASFV receptors, induces antibodies specific to the protein p12 in animals naturally infected and inoculated with inactivated virus or recombinant protein p12, and does not inhibit the binding of virus to host cells or reduce the virus infectivity, indicating that p12 protein mediates the attachment of virions to specific receptors (17). The p54 and p72 proteins are located in the envelope precursors and capsid surface of the virion, respectively. They can neutralize antibodies and block the adsorption of ASFV to macrophages, indicating that p54 and p72 proteins are involved in the adsorption of ASFV (15). The CD2v protein, encoded by the EP402R gene, is a glycoprotein similar to the T lymphocyte surface adhesion receptor CD2v. The CD2v protein is required for mediating erythrocyte surface adsorption and binding of extracellular virions to erythrocytes (22).

**Table 1:** ASFV encodes proteins involved in adsorption and invasion processes.

Viral Proteins	ORFs	Functions
p12	O61R	Involves in virus adsorption ^[17]^
p54	E183L	Involves in virus adsorption ^[15]^
p72	B646L	Involves in virus adsorption ^[15]^
CD2v	EP402R	Involves in binding of erythrocytes to infected cells and extracellular virions ^[30]^
p30	CP204L	Involves in virus intrusion ^[20]^
pE248R	E248R	Involves in viral and endosomal membrane fusion ^[28]^
pE199L	E199L	Involves in membrane fusion and penetration of the core ^[29]^

Virus invasion of host cells is the first step to induce infection, so understanding the specific pathway of virus entry and accurate regulation mechanism is the key to understand the pathogenesis of virus. In early studies, ASFV is considered to enter cells through receptor-mediated endocytosis and transport along the endolysosomal pathway, which dependent on temperature, energy, cholesterol and low pH. In recent studies, classical clathrin-dependent and dynein-dependent endocytosis have been shown to be the main entry pathways of ASFV. In addition, ASFV also enters host cells through macropinocytosis. This is a non-selective endocytosis involving actin-driven membrane fold-mediated uptake in the liquid phase. In general, ASFV enters host cells through two endocytic pathways: macropinocytosis and clathrin mediated endocytosis (CME). These two endocytosis pathways are not opposite, but cooperate with each other, and both need the participation of actin. The study found that the mechanism of multiple endocytic invasion has also been used for Ebola virus, influenza virus and foot-and-mouth disease virus (23). The p30 protein of ASFV is the main inner membrane protein and important antigenic protein, which is abundantly expressed in the early stage of infection and required for viral endocytosis (19).

Macropinocytosis is an actin dependent endocytosis process activated by receptors and kinases such as PAK-1, PI3K, EGFR and Rho GTPase. In addition, macropinocytosis is to form folds or bubbles on the surface of cell membrane, wrap virus particles and form large cytoplasmic bodies, which are endocytosed into the cytoplasm (24). In a study, it was shown that the use of macropinocytosis by ASFV to enter cells requires Na^+^/H^+^ exchanger, activation of EGFR and PI3K, phosphorylation of PAK1 kinase and activation of Rho GTPase Rac1 (12). CME is usually limited to small and medium-sized viruses with a diameter of 50-100 nm (25), but ASFV particles are detected in clathrin coated vesicles (26). During CME, the virus particles first bind to the receptors on the cell membrane and wrap the virus particles by forming pits, so as to quickly form vesicles with a diameter of 85-120 nm (27). The AP-2 junction complex is oligomerized to the membrane with the participation of other auxiliary molecules such as AP180, Eps15 and phosphatidylinositol. Finally, GTPase dynamic cuts down the vesicles. Once in the cytoplasm, the vesicles lose clathrin and produce early endosomes to continue other processes (28). In addition to macrophages and monocytes, ASFV also infects secondary target cells, such as vascular endothelial cells, hepatocytes or epithelial cells. Therefore, by using different entry routes, ASFV may improve its ability to infect different target cells and adapt to the changing conditions of the infection process.

Vesicles sequentially form early endosomes (EE), mature endosomes (ME), and late endosomes (LE) within the cell. LE may fuse with lysosomes (Lys) for degradation (25). Endosomal maturation requires the presence of some lipids, such as phosphoinositides, on the endosomal membrane for the specific incorporation of proteins involved in trafficking and maturation, called Rab GTPases. The study found that this pathway involves the gradual acidification of the endosomal cavity. At the beginning, the pH value of EE is 6.5, the invagination of intraluminal vesicles (ILV) becomes multivesicular body (MVB), and the pH value of LE after maturation is between 6-5. After the fusion of LES and Lys (characterized by lamp1 expression), the pH value decreases to 5-4.5 (29). In the study, it was found that blocking the acidification of the cavity (such as A1 and ammonium chloride) could seriously reduce the infectivity of ASFV (30). The virus particles in the endosome undergo shelling, the inner envelope fuses with the inner body membrane of LE and is transported out, and the viral DNA is released from the core-shell. The pE248R protein of ASFV is a transmembrane polypeptide of inner capsule membrane, which is necessary for the fusion of virus and inner body membrane (20). In addition, pE199L protein is also a transmembrane polypeptide of the inner capsule, but it is required for membrane fusion and penetration of the core (21). The study found that pE199L protein is similar to A16, G9 and J5 in VACV EFC protein, while pE248R protein is similar to L1 and F9. ASFV can encode a fusion mechanism consisting of pE199L and pE248R involved in viral penetration into the cytoplasm. Another study reported that the protein pE248R located in the inner capsule membrane and inner body membrane cholesterol mediates the fusion of virus inner membrane and restricted inner body membrane. This phenomenon confirms the speculation that ASFV has a similar EFC structure (6).

## Viral genome expression and replication

ASFV has a set of host-independent replication and transcription mechanism, but the translation process is host-dependent. Genome replication and transcription depend on many related proteins encoded by ASFV genome (6).

### Replication

The uncoated virus particles reach the replication site in the perinuclear region near the microtubule tissue center (MTOC) and then migrate to the cytoplasm for replication (15). The nuclear phase of viral DNA replication is about 6 hours after infection, but decreased to almost zero at 12 hours after infection (31). pA104R and p10 are important DNA-binding proteins during replication. Meanwhile, pA104R and ASFV topoisomerase II (pP1192R) showed DNA supercoiling activity. In ASFV-infected cells, pA104R is detected from 12 hour post-infection (hpi) and localized to the viral DNA replication site. After siRNA knockdown experiment, it was found that the number of progeny virus, copy number of viral genome and transcription of late viral gene all decreased. This suggests that the pA104R protein plays a critical role in viral DNA replication and gene expression (32). The latest study deleted pA104R from the genome of the highly virulent ASFV-Georgia2010 (ASFV-G) strain and developed a pA104R-deleted recombinant virus (ASFV-G-ΔA104R) (33). Moreover, animal experiments were performed. It was found that compared with animals vaccinated with ASFV-G, animals surviving with ASFV-G-ΔA104R developed viremia with reduced virus titers, and all animals developed strong virus-specific Antibody responses suggest that pA104R deletion can be used as a live attenuated vaccine candidate. In ASFV-infected cells, p10 protein accumulates in the nucleus at a later stage after infection, suggesting that p10 protein may play an important role in the nucleus at a later stage of the viral replication cycle (34). The pp220 (pCP2475L) protein is a part of the nucleocapsid of ASFV. It can promote the packaging of virus core and inhibit its coding gene, which will lead to the formation and excretion of empty virus particles (35). Furthermore, pp220 is a precursor protein of p150, p37, p34, p14 and p5, wherein both p34 and p14 proteins have nuclear transport activities. The p37 protein is accompanied by viral DNA from the cytoplasm into the nucleus and accumulates in the nucleus. After replication in the nucleus, they are transported to the cytoplasm and accumulated in VF (15, 36). Differently, the p14 protein accompanies the viral DNA into the nucleus but is not involved in the process from the nucleus to the cytoplasm (37). The C962R gene of ASFV encodes NTPase similar to poxvirus D5 protein, which plays a role in the initiation of DNA replication and changes the pattern of DNA replication (38). The pF1055L protein is similar to herpes virus UL-9 protein. The UL-9 protein is involved in binding to the origin of replication and fused with a putative DNA primerase (39), so the pF1055L protein may be involved in the initiation of DNA replication. Similarly, G1211R, E301R and C962R encode DNA polymerase B, PCNA-like protein, and nucleoside triphosphatase, respectively, and are also involved in the initiation of DNA replication (40). By detecting the mRNA levels of two RNA helicases encoded by ASFV pQP509L and pQ706L, it is speculated that they are involved in the replication and transcription of genes in the middle and late stages of ASFV, and have non-redundant roles (41). The pP1192R protein is also known as type II DNA topoisomerase (Topo II). In the middle and late stage of ASFV infection, Topo II is expressed in the cytoplasm of porcine macrophages and transported to VF to participate in ASFV replication. It is actively transcribed throughout infection, and transcripts are detected as early as 2 hpi, reaching peak concentrations around 16 hpi, when viral DNA synthesis, transcription, and translation are more active (42). And in this study, it was found that the addition of enrofloxacin from 15 to 16 hpi induced fragmentation of the viral genome, while the viral genome was not detected when enrofloxacin was added from 0 to 2 hpi, indicating that fluoroquinolones are ASFV-Topo II poison (42). In addition, coumermycin A1, doxorubicin and amsacrin also had a significant effect on its expression (43). The protein can effectively dissociate the kinetoplast DNA (kDNA) and gradually relax the supercoiled structure of DNA (43).

Macrophages are very rich in free radicals, which will cause sustained damage to the virus genome, such as strand breaks and spontaneous depurification or depyrimidine, resulting in DNA damage and hindering the proliferation of ASFV in macrophages (44). In order to effectively overcome these DNA damage, ASFV has evolved its own repair system: ASFV repairs DNA damage through base excision repair (BER). ASFV-encoded ATP-dependent DNA ligase (pNP419L), Pol X (pO174L) and AP endonuclease (pE296R) are important components in the DNA base excision repair pathway. They can correct the DNA damage induced by host macrophages in hyperoxia environment and ensure the integrity of ASFV genome (40).

Many large DNA viruses encode enzymes involved in nucleotide metabolism that increase the dNTPs required for viral DNA replication. ASFV encodes thymidylate kinase (pK196R) and uracil deoxyribonucleoside triphosphatase (pE165R), and their deletion significantly reduces replication in macrophages (40, 45). In addition, ASFV also encodes thymidine kinase (pA240L) and two ribonucleotide reductase subunits (pF134L, pF778R), which are involved in nucleotide metabolism and provide energy for virus replication (46). It is worth mentioning that the dUTP enzyme (pE165R) ensures high fidelity of genome replication and can reduce the chance of misincorporation of deoxyuridine into viral DNA at low dUTP concentrations (**Tables 2**–**4**) (40, 46, 47).

**Table 2:** ASFV encodes related proteins involved in the genome duplication process.

Viral Proteins	ORFs	Functions
pA104R	A104R	Involves in viral DNA replication (32)
p10	K78R	Functions in the nucleus at the late stage of viral replication (34)
p34	–	From pp220 protein hydrolysis, accompanied by viral DNA transport (15, 36)
p14	–	From pp220 protein hydrolysis, accompanied by viral DNA into the nucleus (37)
DNA primase	C962R	It plays a role in the initiation of DNA replication and changes the pattern of DNA replication (6)
pF1055L	F1055L	Involves in the initiation of DNA replication (39)
DNA polymerase family B	G1211R	Involves in the initiation of DNA replication (40)
Proliferating cell nuclear antigen (PCNA)-like pE301R	E301R	Involves in the initiation of DNA replication (40)
RNA helicase	QP509L	Participates in the replication and transcription of metaphase and late gene of virus (41)
RNA helicase	Q706L	Participates in the replication and transcription of metaphase and late gene of virus (41)
Topo II	P1192R	Dissociates kDNA and gradually relaxes DNA supercoiled structure (43)
ATP-dependent -DNA ligase	NP419L	They are important components of BER, repair DNA damage and ensure the integrity of the ASFV genome (40)
DNA polymerase type X	O174L	
AP endonuclease	E296R	
Thymidine kinase	K196	Involves in nucleotide metabolism, increasing dNTPs required for viral DNA replication (40, 45)
dUTPase	E165R	Involves in nucleotide metabolism, increases dNTPs required for viral DNA replication, and ensures high fidelity of genome replication (40, 46, 47)
Thymidylate kinase	A240L	Involves in nucleotide metabolism, increasing dNTPs required for viral DNA replication (46)
pF134L	F134L	Ribonucleotide reductase subunit, involved in nucleotide metabolism to increase dNTPs required for viral DNA replication (46)
pF778R	F778R	

**Table 3:** ASFV encodes related proteins involved in genome transcription.

Viral Proteins	ORFs	Functions
RNA polymerase subunit 1 (pNP1450L)	NP1450L	Involves in viral gene transcription (40)
RNA polymerase subunit 2 (pEP1242L)	EP1242L	Involves in viral gene transcription (40)
RNA polymerase subunit 3 (pH359L)	H359L	Involves in viral gene transcription (40)
RNA polymerase subunit 5 (pD205R)	D205R	Involves in viral gene transcription (40)
RNA polymerase subunit 6 (pC147L)	C147L	Involves in viral gene transcription (40)
RNA polymerase subunit 7 (pD339L)	D339L	Involves in viral gene transcription (40)
RNA polymerase subunit 10 (pCP80R)	CP80R	Involves in viral gene transcription (40)
Transcription factor SII	I243L	Starts the transcription of virus gene from different sites (48)
TFIIB like	C315R	Forms a transcription initiation complex with RNA polymerase to initiate viral gene transcription (49)
pB263R	B263R	Involves in viral gene transcription (50)
pA859L	A859L	Helicase protein, involves in viral gene transcription (40)
pF105L	F105L	
pB92L	B92L	
pD1133L	D1133L	
pQ706L	Q706L	
pQP509L	QP509L	
Guanylyl transferase	NP868R	Adds cap structure and poly A tail to the 5’ and 3’ ends of ASFV mRNA (51)
NUdix hydrolase	D250R	Involves in the cleavage and hydrolysis of mRNA caps and mRNA degradation, regulating the transcription of viral genes (52)
PolyA polymerase large subunit	C475L	Involves in mRNA processing (40)
FTS-J-like RNA methyltransferase	EP424R	Involves in mRNA processing (40)

**Table 4:** ASFV encodes related proteins involved in genome translation.

Viral Proteins	ORFs	Functions
pDP71L	DP71L	Binds to PP1 to dephosphorylate eIF2α and enhance viral protein synthesis (6, 53)
pA224L	A224L	Inhibits protease caspase-3 to ensure viral protein translation and synthesis (54, 55)
FTS-J-like RNA methyltransferase	EP42R	Stabilizes rRNA in cells and prevents disruption of viral protein synthesis (6)

### Transcription

In the cytoplasm of infected cells, ASFV mainly relies on a variety of self-encoded enzymes and transcription factors to regulate the transcription of viral genes, while the RNA polymerase II of host cells is not involved in this regulatory process (40, 56). Gene transcription, coordinated with DNA replication, is the main switch for ASFV gene expression. Immediate early and early genes are expressed within 4-6 hours after virus infection, which before the beginning of DNA replication, after ASFV infection for 8-16 h, the intermediate and late genes begin to be expressed, but intermediate and late genes are expressed later (15). Up to now, it has been found that the RNA polymerase subunits that may be involved in ASFV mRNA synthesis mainly include pNP1450L (RP1), pEP1242L (RP2), pH359L (RP3), pD205R (RP5), pC147L (RP6) and pD339L (RP7) and pCP80R (RP10) (40). ASFV also encodes specific transcription factors, such as transcription factor TFIIS (pI243L) and transcription factor-like TFIIB (pC315R). The pI243L protein can initiate transcription from different sites, resulting in early, middle and late mRNAs, with relatively low levels of early mRNAs and high levels of late mRNAs (48). However, the pC315R protein cooperates with RNA polymerase to form a transcription initiation complex and initiate viral gene transcription (49). In addition, the B263R-encoded protein of ASFV has the characteristics of TATA-binding protein and may be involved in viral gene transcription (50). Vaccinia virus (VACV) and ASFV belong to nucleocytoplasmic large DNA viruses (NCLDV) (39). Although VACV and ASFV are different in morphology, they have many common biological characteristics, such as genome structure, replication, and transcription, which are almost completely independent of the host cells (57). Therefore, VACV proteins can provide research clues for the helicase protein encoded by ASFV. ASFV encodes six helicase proteins, namely pA859L, pF105L, pB92L, pD1133L (g10L), pQ706L (j10L) and pQP509L (j11L) (40). Among them, pD1133L, pQ706L and pQP509L proteins are involved in ASFV gene transcription. The pD1133L and pQ706L proteins have the highest homology to the D11L protein of VACV (58). The D1133L and B962L genes are located in the central region of the ASFV genome. In the late stage of ASFV infection, after viral DNA replication is initiated, these two genes can be expressed in Vero cells cultured *in vitro* (59).

ASFV transcripts are modified in the cytoplasm by adding a 5’-capping and a 3’-polyadenylation. ASFV encodes a guanylate transferase (pNP868R) with three functional domains, namely triphosphatase (TPase), guanosyltransferase (GTase) and methyltransferase (MTase), which can be located at the 5’ end of ASFV mRNA and 3’ ends to add cap structure and poly(A) tail, respectively (51). The g5R protein (D250R), also known as diphosphate nucleotide hydrolase, is encoded by the D250R gene, expressed in the early stage of viral infection, co-localizes with the mRNA cap structure, participates in the cleavage and hydrolysis of mRNA caps and mRNA degradation, and regulates viral gene transcription (52). In addition, poly A polymerase (pC475L) and pEP424R proteins encoded by ASFV were involved in mRNA processing (40).

### Translation

Due to the limited size of the viral genome, it relies on host cells translation machinery to efficiently synthesize its proteins (60). During ASFV infection, cellular protein synthesis is strongly inhibited, whereas viral proteins are efficiently produced. ASFV can hijack eukaryotic initiation factor (eIFs) including eIF2, eIF4F, eIF4G and eIF4E to complete the synthesis of its proteins (52). The ASFV pDP71L, pA224L and pEP424R proteins are known to regulate the viral translation pathway. In eukaryotic cells, eukaryotic initiation factor 2α (eIF2α) is a critical and rate-limiting step in the regulation of overall protein synthesis (61). The pDP71L protein of ASFV can bind to host-encoded phosphatase 1 (PP1) and dephosphorylate eIF2α, which can enhance viral protein synthesis (6, 53). Interestingly, deletion of DP71L from one isolate of ASFV (E70) reduced viral virulence, whereas no reduction in viral virulence was observed from another isolate (Malawi LIL20/1). The reason for this difference may be the influence of other genes (62). Eukaryotic initiation factor 4F (eIF4F) consists of eIF4E, eIF4A, and eIF4G, and eIF4F plays an important role in cellular mRNA translation (63). The ASFV pA224L protein is a member of the inhibitor of apoptosis protein (IAP) family. In the process of apoptosis, eIF4G is cleaved by the cellular protease caspase-3, but pA224L protein can inhibit the protease to ensure the translation and synthesis of viral proteins (54, 55). The ASFV pEP424R protein is an FTS-J-like RNA methyltransferase that stabilizes rRNA in cells to prevent disruption of viral protein synthesis (40).

In addition to the above viral proteins involved in the expression and replication of the ASFV genome, it is also worth mentioning the pI215L protein. pI215L protein, also known as E2 ubiquitin-binding enzyme, is homologous to ubiquitin-binding protein and involved in ASFV replication and transcription (64, 65). The pI215L gene is transcribed from early infection, showing two major transcriptional peaks (2 and 16 hpi), suggesting that pI215L may be involved in different stages of the viral life cycle (64). At 2-4 hpi of Vero cells, the pI215L gene initiates transcription and translation, respectively; at 8h after infection, the protein is transported to VF and evenly distributed in the nucleus and cytoplasm during virus infection. The pI215L protein can bind to eIF4E, which affect the mammalian target protein of rapamycin (mTOR) signaling pathway and regulate the expression of host cell proteins (66).

## Mature virus particles assembly and progeny virus release

ASFV, like poxviruses and iridoviruses, is assembled in the VF close to the MTOC (67, 68). MTOC contains viral DNA, most viral proteins, immature and mature virus particles, and abundant membrane structures (69). The formation of VF requires the integrity of microtubules, and the transport of virus particles is associated with stable microtubules (70). Noridazole interferes with the polymerization of microtubule filaments and prevents the formation of VF (69, 71). The fully formed VF is a single structure, lacking an external limiting membrane and surrounded by mitochondria and vimentin cage. Vimentin cage can provide a physical scaffold for VF or prevent viral components from entering the cytoplasm (16, 72). ASFV assembles in the VF around the host cytoplasmic nucleus, and the viral assembly sites are concentrated in the microtubule formation center and Golgi complex (73). Firstly, the capsid and nucleocapsid domains of the virus are assembled to form the precursor of icosahedral (74). The last step of virion morphogenesis will be the encapsidation of DNA giving rise to mature virions. Mature virions rely on kinetokinase and ASFV capsid proteins to move from the VF to the cell membrane and leave the host cells by budding (16). Besides, there are many viral proteins involved in this process (**Table 5**).

**Table 5:** ASFV encodes related proteins involved in the assembly and release process.

Viral Proteins	ORFs	Functions
p10	K98R	Involves in viral genome packaging (47)
pA104R	A104R	Involves in viral genome packaging (47)
pp220	CP2475L	Facilitates viral core packaging (47, 75)
pp62	CP530R	Corrects the assembly and maturation of viral nucleoid core (47, 76)
p72	B646L	Affects the normal expression of pp220 and pp62, forming the capsid of the virus particle (35, 77)
SUMO-1-like protease	S273R	Involves in the hydrolysis of pp220 and pp62 proteins (78)
p54	E183L	Binds specifically to LC8 and involves in the transport of virions (79)
p17	D117L	Promotes the formation of viral dodecahedral particles (80)
p49	B438L	Facilitates the formation and stabilization of virion dodecahedral vertices (81, 82)
p14.5	E120R	Together with the p72 protein, they form the capsid of the virus particle (83)
pB602L	B602L	Assists in the assembly of p72 proteins (40)
Trans-prenyl transferase	B318L	Involves in VF and/or virus assembly (6)

The ASFV genome is protein-wrapped to form the viral nucleoid. Immunoelectron microscopy showed that two DNA-binding proteins, p10 (pK78R) and pA104R, are located in the nucleoids of mature virions, possibly playing a role in the assembly of viral nucleoids (47). The polyprotein hydrolyzate is mainly located in the inner core shell of the virion, accounting for about 30% of the total viral proteins, and is the main component of the inner core shell. The pp220 and pp62 proteins are ASFV polyprotein precursors, encoded by the CP2475L and CP530R genes, respectively (47). The hydrolysis of pp220 and pp62 proteins are carried out at the same time as the assembly of virus particles. They can be cleaved into mature viral proteins by proteolytic hydrolysis and assembled together into the DNA-containing nucleoid core shell (35, 83). The processing of pp220 and pp62 proteins depends on the expression of ASFV capsid protein p72. When the expression of p72 protein is blocked, the unprocessed poly proteins pp220 and pp62 will assemble into an abnormal zipper like structure, and the processing of pp62 requires the expression of pp220 precursor (35). In addition, these two proteins also formed zipper-like structures when co-expressed in COS cells (35). The pp220 protein is an N-myristoylated precursor polypeptide, and its myristoyl moiety can act as a membrane anchoring signal, which can bind the developing core-shell to the inner envelope of the virus and serve as an internal nucleoplasm A protein scaffold between the body and the outer nuclear layer that drives the assembly of the empty shell, thus pp220 is an essential component of core assembly (75). The study found that the virus-encoded SUMO-like S273R protease is involved in the hydrolysis of polyproteins (78). The proteolytic processing of pp220 produces p150, p37, p34 and, p14 and p5 proteins. Among them, p34 and p150 protein membrane-related components can be assembled into viral matrix structures, most of the misprocessed p150 can also be recovered from the cytosol, and the correctly processed p150 products are selectively aggregated to the inner capsule membrane (84). The proteolytic processing of pp62 produces p35, p15 and p8 proteins, which are late expression proteins of viral DNA replication, and inhibition of their expression results in the appearance of mostly empty virions (76). It is found that the structural protein P54 in ASFV specifically interacts with the cytoplasmic dynamic protein light chain (LC8) during viral infection, which may be involved in the process of microtubule virus transport (79). Furthermore, p54 and LC8 interacted both *in vitro* and *in vivo*, and both co-localized in the MTOC during ASFV infection. The inner envelope protein that participates in the assembly of the virus is also the p17 protein. A late membrane protein expressed by ASFV, p17 is a transmembrane protein on the viral inner envelope, which facilitates the further assembly of the icosahedral intermediate in the viral precursor membrane and is essential for viral viability (83). When p17 gene expression is inhibited, the proteolytic processing of pp220 and pp62 is blocked, which can lead to the assembly of the virus into core defective icosahedral particles (80).

The viral capsid protects viral nucleic acids from damage by nucleases or other physicochemical factors in the environment. Currently, proteins p72 (pB646L), P49 (pB438L) and P14.5 (pE120R) are known to play important roles in the formation of ASFV core shell or capsid. The p72 protein assembly requires the assistance of the B602L-encoded molecular chaperone (40). In the early stage of virion formation, p72 is enriched in the cytoplasm and binding to the endoplasmic reticulum membrane to form the virion capsid on the convex surface of the endoplasmic reticulum membrane and the inner core shell on the concave surface (77). With the maturation of virus, the antioxidant capacity of the p72 protein is enhanced, which may be in preparation for the release of the virus (28). The p49 protein is expressed in the late stage of ASFV infection. In the absence of this protein, the assembled virions do not have an icosahedral symmetrical structure, but an abnormal tubular structure (81). Under electron microscope observation, it is found that this protein is located near the apex of the capsid, indicating that this protein plays an important role in the construction or stabilization of the icosahedral apex of virus particles (47). During viral infection, the p49 protein binds to the membrane and appears as an intact membrane protein (81, 82). In addition, the p14.5 protein is also expressed in the late stage of ASFV infection, and p14.5 and p72 together constitute the capsid of the virus particle (83). Besides, the p14.5 is involved in the intracellular transport of virions, which plays an important role in the formation of the inner core shell and capsid of the virus (85). In addition, the trans-pentenyl transferase encoded by the B318L gene in ASFV localizes to the viral assembly site, associates with the precursor viral membrane from the endoplasmic reticulum, and may play a role in VF and/or viral assembly (6).

Mature virus particles rely on kinesin and ASFV capsid protein, which are transported from VF to the cell surface through microtubule channel. Finally, they are discharged out of the cells by budding and obtain an outer membrane (16, 79, 85).

## Discussion

ASFV, which is a Nucleocytoplasmic Large DNA Virus (NCLDV), belongs to the family of *Asfarviridae*. Due to the huge genome, complex structure and multiple genotypes, there is still no effective commercial vaccine against ASFV (6). The replication cycle of ASFV includes viral attachment, internalization, genome proliferation, assembly and release of progeny viruses. The ASFV genome encodes 150-200 viral proteins, which play important roles in many aspects of the viral infection cycle.

The proteins encoded by ASFV can act on the viral replication cycle in various ways. For example, in the process of ASFV adsorption to host cells, the p12 protein allows cell surface membrane proteins to act as ASFV receptors, indicating that this protein mediates the attachment of virions to specific receptors (17). Previous studies has shown that CD163 is considered to be a potential ASFV infection receptor, but in subsequent studies it was found that CD163 is not sufficient for the virus to infect host cells (86, 87). Therefore, whether there is receptor mediated ASFV infection and what the specific receptor is need to be further studied in the future. Both p37 and pP1192R proteins are transported to VF and accumulated during viral replication (43, 64). And the trans-pentenyl transferase (pB318L) is associated with the precursor viral membrane from the endoplasmic reticulum and may play a role in VF and/or viral assembly (6), but the mechanism of VF membrane structure formation is also unclear.

Scientific research must ultimately serve life, but there is no commercial vaccine that can prevent and control African swine fever. It is of great significance to research on key proteins that participate in ASFV replication or infection, such as p54, p30, pp220, pp62, p72, CD2v, etc. These proteins are considered immunogenic proteins that can be used to construct subunit vaccines.

In summary, although the research on related ASFV has been deepened in recent years, there are still some questions that may be worth studying. Are there other encoded viral proteins involved in viral replication? How do encoded proteins with similar functions coordinately regulate viral replication? Which viral proteins interact with host cell proteins and the specific mechanisms of action remain unclear. This series of problems will definitely become the hotspot of ASFV research. The solution of these problems will provide a theoretical basis for its vaccine research and development and antiviral drug preparation and will provide ideas for formulating corresponding diagnostic schemes and prevention and control strategies.

## Author Contributions

XD, HZ, and YR conceived and designed the study. XD, YR, WY, JR, RH, XQ, DL, and HZ wrote the manuscript. All authors contributed to the article and approved the submitted version.

## Funding

This work was supported by the National Key Research and Development Program of China (2021YFD1800100), and grants from the Gansu major science and technology projects (20ZD7NA006 and 21ZD3NA001-5), Luoyang city Science and Technology Major Project (Y2019YJ07-01), Development of African swine fever subunit vaccine(CAAS-ASTIP-JBGS-20210401).

## Conflict of Interest

The authors declare that the research was conducted in the absence of any commercial or financial relationships that could be construed as a potential conflict of interest.

## Publisher’s Note

All claims expressed in this article are solely those of the authors and do not necessarily represent those of their affiliated organizations, or those of the publisher, the editors and the reviewers. Any product that may be evaluated in this article, or claim that may be made by its manufacturer, is not guaranteed or endorsed by the publisher.
